# Communication Skills for Patient Engagement: Argumentation Competencies As Means to Prevent or Limit Reactance Arousal, with an Example from the Italian Healthcare System

**DOI:** 10.3389/fpsyg.2016.01472

**Published:** 2016-09-27

**Authors:** Sarah Bigi

**Affiliations:** Department of Linguistic Sciences and Foreign Literatures, Catholic University of the Sacred HeartMilan, Italy

**Keywords:** psychological reactance, deliberation dialogue, patient engagement, adherence, patient education

## Abstract

The paper discusses the role of argumentative competencies for the achievement of patient engagement through communication in doctor-patient consultations. The achievement of patient engagement is being proposed by recent studies as a condition that can facilitate in particular patient adherence, which involves behavior change. One obstacle to behavior change that has been observed is reactance, i.e., resistance to persuasive messages when a threat to freedom is perceived. In the medical field, reactance theory has been mostly applied in the field of mental health, less frequently to understand non-adherence in general. However, a few studies have revealed that reactance can actually explain in part the motives behind non-adherence. These studies propose that the arousal of reactance could be limited or prevented by adopting relational measures aimed at giving patients the feeling that they still hold some control over the process of care and that the “impositions” on their freedoms are acceptable because they have had the opportunity to decide about them. However, they do not discuss how these strategies should be operationalized at the dialogical level. A debated issue in the study of reactance is the role played by knowledge. It seems that pure information regarding an issue is likely to represent a threat in itself. Complementary to this is the finding that quality of argument does not impact on the degree of reactance. These findings pose a problem in view of the goal of patient education, itself considered as a necessary premise for any process of patient engagement and adherence. It seems necessary to move away from a conception of education as mere transmission of information and look for more effective ways of transferring knowledge to patients. With regard to this issue, the paper argues that useful insights can be found in studies on science education, in which it is shown experimentally that argumentative processes favor learning and understanding. Drawing on previous studies and taking an interdisciplinary perspective on the issue, the paper brings into the discussion on engagement concepts developed in the field of argumentation theory, showing how the suggestions for avoiding reactance could be realized dialogically.

## Introduction

The paper discusses the role of argumentative competencies for the achievement of patient engagement through communication in doctor-patient consultations.

Patient engagement “qualifies the relation that the patient […] may establish with his/her reference healthcare system […] in the different phases of the care process” (Graffigna et al., 2015, p. 8). It is a concept borrowed from the marketing and consumer behavior literature to describe consumers' positive attitudes toward brands or products (Gambetti and Graffigna, 2010). In the healthcare context, it is meant to signify a proactive attitude of patients, understood as “consumers” of healthcare. Such engaged attitude, when successfully achieved, would imply that patients are proactive on two levels: (1) they are able to correctly solicit the healthcare system when in need for assistance; (2) they are able to correctly manage their health condition without improperly referring to the healthcare system (patient autonomy).

In this sense, the concept of engagement takes a step forward in comparison to the notions of compliance, adherence, self-management, patient empowerment and patient activation, as it refers to the relationship between patients and healthcare systems in their complexity. Moreover, it implies a more active view of patients, who are not simply “activated” by their providers, but independently take an active role in the management of the whole process of care that concerns them (Graffigna et al., 2015, pp. 16–20).

**Figure 1:**
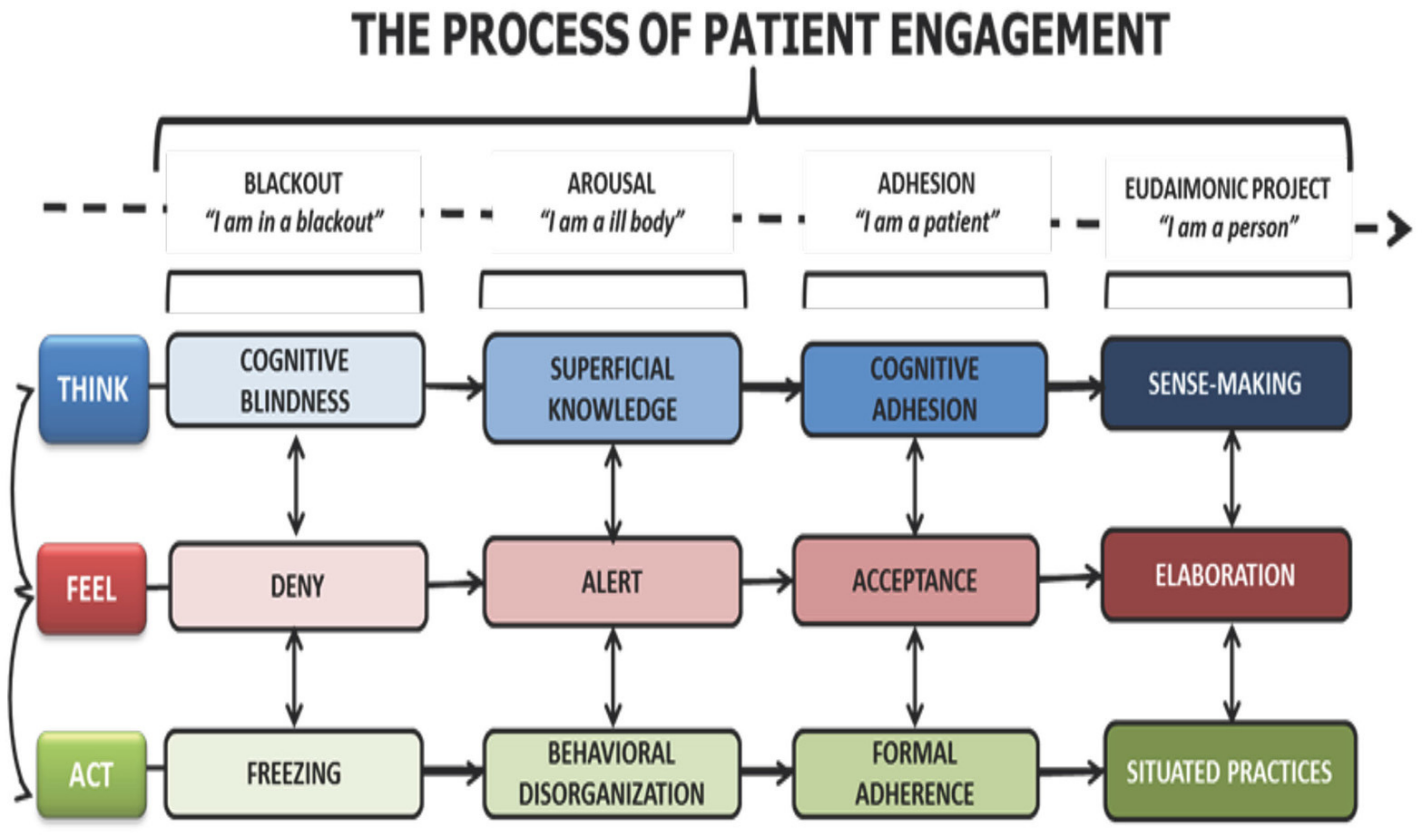
**The Patient Health Engagement Model**.

Full patient engagement is the result of a gradual process that allows patients to rise from a condition of blackout, to one of eudaimonic project, in which the disease is not the center of attention anymore, but is fully integrated in the patient's life (Graffigna et al., 2014). In other words, in the eudaimonic project condition (the “perfection” of engagement) patients do not perceive themselves just as “patients,” but as individuals who also have a health condition that requires some attention. But the actions that need to be taken in order to manage this health condition are not felt as impairing as they might have been at the beginning (Graffigna et al., 2014, 2015). In the Patient Health Engagement Model (PHE), Graffigna and colleagues have detailed the phases patients go through in their progress toward the condition of eudaimonic project. The Model is represented in Figure 1.

The PHE Model details and relates the cognitive, emotional and behavioral conditions that characterize the pathway from the initial condition of blackout, usually following the first diagnosis, to the condition of being fully engaged and able to integrate the health condition within a life project. As full engagement is the final outcome of this progress, it appears that, especially at the beginning but also along the way, individuals need to be educated to understand their new status as patients, and motivated to take up any new behavior that their health condition requires (Graffigna et al., 2015, pp. 36–38). Thus, it appears that the condition of “being engaged” originates in the one-to-one relationship with healthcare providers (medical doctors, nurses, counselors, etc.) and is triggered by their ability to motivate patients and support their efforts toward behavior change.

Particularly in this respect, “being engaged” should include among other behaviors the ability for patients to more easily adhere to providers' therapeutic prescriptions and suggestions for healthier lifestyles (Graffigna et al., 2014). As such, engagement is understood as a pre-requisite for adherence (Graffigna et al., 2015, p. 17).

In the following discussion, attention is devoted in particular to the communicative processes that may play a role in the activation of patient engagement. The assumptions on which the discussion is based are that: (1) patient engagement originates in the relationship with one or more healthcare providers in their capacities as representatives of the healthcare system; (2) the tools that support and maintain this relationship are communicative in nature. More specifically, the paper discusses the case of psychological reactance, which can hinder motivation to behavior change and engagement. As such, reactance can be considered as one of the many judgment biases that have been identified by researchers in the behavioral sciences during the past 40 years or so (Fischhoff, 1975; Kahneman et al., 1982; Petty and Cacioppo, 1986). For many of these biases, corresponding debiasing techniques have been studied and tested (Fischhoff, 1982; Arkes, 1991; Lewandowsky et al., 2012; Croskerry et al., 2013). Also drawing on previous studies (Bigi, 2014a, 2015; Bigi and Lamiani, 2016), it is argued that reactance can be prevented or limited by an appropriate use of argumentative strategies, used within the structure of the deliberation dialogue. In this sense, argumentation strategies could be considered and should be further experimentally tested as a form of debiasing.

## Psychological reactance in relation to the problem of non-adherence

Psychological reactance is described as resistance to persuasive messages when a threat to freedom is perceived (Brehm, 1966; Brehm and Brehm, 1981). This condition brings individuals to do the contrary of what they are asked to do or to persist in a wrong behavior even in the face of evidence. The key factors involved in the arousal of reactance are freedom, threat to freedom, reactance and restoration of freedom. Studies have shown that reactance is the result of a combination of cognitive and affective processes, in which negative cognition and anger play a major role (Dillard and Shen, 2005; Rains and Turner, 2007).

The analysis of reactance as a potential threat to the achievement of patient engagement is relevant for two orders of reasons: (1) the institutional and asymmetrical nature of interactions between patients and providers, which could imply in itself a threat to freedom; (2) the fact that little attention has been paid so far to the role reactance could play in relation to non-adherence.

In the medical field, reactance theory has been mostly applied in the field of mental health, less frequently to understand non-adherence in general (Fogarty, 1997). However, a few studies have revealed that reactance can actually explain in part the motives behind non-adherence (Fogarty, 1997; Dillard and Shen, 2005; Orbell and Hagger, 2006; Rains and Turner, 2007). These studies propose that the arousal of reactance could be limited or prevented by adopting certain measures, e.g., spending time with patients familiarizing them with the procedures they will undergo, answering their questions and listening to their non-medical comments; simplifying the behavior changes requested of patients; reducing the magnitude of the requested tasks; when possible, offering the opportunity to choose among different and equally acceptable solutions to the problem (Fogarty, 1997). These and other strategies are aimed at giving patients the feeling that they still hold some control over the process of care and that the “impositions” on their freedoms are acceptable because they have had the opportunity to decide about them. However, these studies do not discuss how these strategies should be operationalized at the communicative level, and more specifically at the level of dialogue. In particular, it is not clear what role is played by knowledge and persuasion.

As far as knowledge is concerned, it seems that pure information regarding an issue, e.g., a disease, is likely to represent a threat in itself, as it sheds light on possible limitations to freedom for the individual, who in turn will experience reactance (Brehm and Brehm, 1981; Fogarty, 1997). In particular, experimental findings (Dillard and Shen, 2005) suggest that those who design health messages should pay attention to health topics that may interfere with strength of threat: the study reported an experiment with messages related to flossing vs. responsible drinking, targeting college students. The latter were found to arouse more reactance because they were perceived as more threatening of freedom. The problem is that responsible drinking runs counter to social norms of conduct in certain groups, therefore complying with messages suggesting different styles of drinking puts individuals at risk of being stigmatized within their social group (Dillard and Shen, 2005, pp. 163–164). These findings suggest that the role and provision of information to patients deserves careful consideration especially at the dialogical level.

With regard to persuasion, another study found that quality of argument does not impact on the degree of reactance (Rains and Turner, 2007), because it seems that perceiving a threat to freedom is enough to cause anger and negative cognitions that actually make the quality of arguments irrelevant. In other words, once certain information has been provided and reactance has been aroused, the potential benefits of persuasion are reduced by the emotional component of reactance itself, i.e., anger and negative cognition. It also seems that reactance is strengthened by the perception of dominance, i.e., the extent to which a message reveals that the sender believes s/he can control the receiver. On the other hand, when justifications for requesting a certain behavior are provided, this softens the feeling of threat and reduces the arousal of reactance. It is possible to distinguish in these findings two different ways in which argumentation is used: with the aim of persuading (in the sense of obtaining consensus or compliance) and with the aim of providing reasons to support a claim (with the aim of finding agreement over a debated issue).

In all the studies reported, there seems to be a close connection between the arousal of reactance and the processes of providing information about and reasons for action. In particular, it seems that the quantity, quality and context of the messages that are conveyed to patients can dramatically change the way threats to freedom are perceived. It appears therefore justified to try and understand the informative and argumentative processes from a dialogical perspective, which may allow to clarify their structures and roles in relation to reactance on the one hand, and engagement on the other. To this end, in the following sections insights from the field of argumentation theory are called into the picture and discussed in relation to the notion of reactance.

## Preventing reactance arousal through effective pragmatic argumentation

In her discussion on reactance and patient non-adherence, Fogarty (1997, pp. 1282–1283) puts forward a series of practical suggestions that should limit patients' reactance to providers' indications.

The final goal of providers' efforts at patients' involvement is, in Fogarty's terms, fueling patients' perception that they are retaining some degree of control over the procedures they need to undergo, and that they are freely conceding something to the provider, instead of being persuaded against their will into something they did not want to do. This should decrease the perception of loss of freedom, thereby also reducing reactance. The process of eliciting patients' cooperation should also increase the likelihood of future adherence, as studies show that those who comply with small requests are more likely to comply again, even with larger requests, in the future (Freedman and Fraser, 1966; Snyder and Cunningham, 1975; Souchet and Girandola, 2013). By explicitly requesting patients' cooperation at the beginning of the encounter, Fogarty suggests that an atmosphere of “mutual interdependence” (1997, p. 1283) is created, which can be preserved by letting patients become active participants in the discussion regarding therapeutic regimens, so that the final decision will result in a “negotiated regimen” formulated through the integration of patients' perspectives. In this respect, Fogarty provides a list of practical suggestions for providers:

1. discuss with patients how to fit the prescribed regimen into their and their families' lifestyles;
2. keep treatments as simple as possible (on this concept, see also more recent experiments by Fogg, http://tinyhabits.com);
3. show willingness to keep therapies as short as possible and to eliminate unnecessary proposals;
4. offer more than one effective alternative, whenever possible, and let patients select the one that best fits their preferences and possibilities. This suggestion in particular is aimed at communicating the perception of the provider as someone who is willing to make concessions. A universal rule of behavior (Cialdini, 2007; Ariely, 2008) dictates that when one party is willing to make concessions, the other one will reciprocate.

The kind of “discussion” described by Fogarty, basically corresponds to what argumentation scholars call “pragmatic argumentation,” which happens when the parties need to agree on the solution to a problem, and discuss the validity of a course of action based primarily on its consequences (Perelman, 1959). In this use of argumentation, the positive or negative evaluation of the consequences is transferred to the causes, which are accordingly accepted or rejected.

In more concrete terms, during the medical encounter one therapeutic regimen may be preferred over another because it is believed to obtain more positive consequences. Clearly, in order to align each other's criteria for the evaluation of the consequences it is necessary for both parties to be able to express their preferences during the discussion. Moreover, in order to put into practice suggestions 1. and 2. patients should be able to put forward their own proposals or perspectives on the provider's suggestions. In order for this communicative process to be effective, it cannot be left entirely to the good will or talent of providers. Its inner workings, potential and risks should be laid out and explained. In the next section, this is done by resorting to the model of the deliberation dialogue (Walton, 1989; Walton and Krabbe, 1995).

## Advantages of using a model of deliberation

The model of the deliberation dialogue has been developed within an approach that aims at representing and analyzing types of dialogue as communicative intentions within a verbal interaction (Walton and Krabbe, 1995; Walton and Macagno, 2007). As such, the representations of the types of dialogue are abstract, normative frameworks capturing shared dialogical intentions. The deliberation dialogue is one among seven types of dialogues, described according to the intentions of and the initial relationship between the interlocutors: information-seeking, persuasion, deliberation, inquiry, negotiation, and eristics.

The structure of the deliberation dialogue outlines the most effective dialogical moves aimed at finding an acceptable course of action to achieve a certain goal (Walton, 2010; Walton et al., 2010). This kind of dialogue usually takes place when there is no compelling objective way of coping with a problem and parties discuss their reasons for proposing a certain solution; in this sense, it is a representation of pragmatic argumentation. It is therefore appropriate for the representation of the deliberative process occurring within a medical encounter when providers and patients need to discuss the acceptability of a therapeutic regimen or of a specific behavior.

One important premise for deliberation dialogues is that parties are out to reach a collective goal, which can be contrary to or different from the individuals' personal goals (Walton et al., 2010). This is true also of the kind of deliberations occurring within medical encounters. Indeed, the arousal of reactance can be partly explained as resistance to a potential threat that is perceived due to a misalignment between the parties' intentions and preferences. Part of the deliberative effort is precisely to set a shared goal in a collaborative way (Bigi, 2014a), which is basically what Fogarty (1997) suggests when proposing that patients' collaboration be explicitly invited.

**Figure 2:**
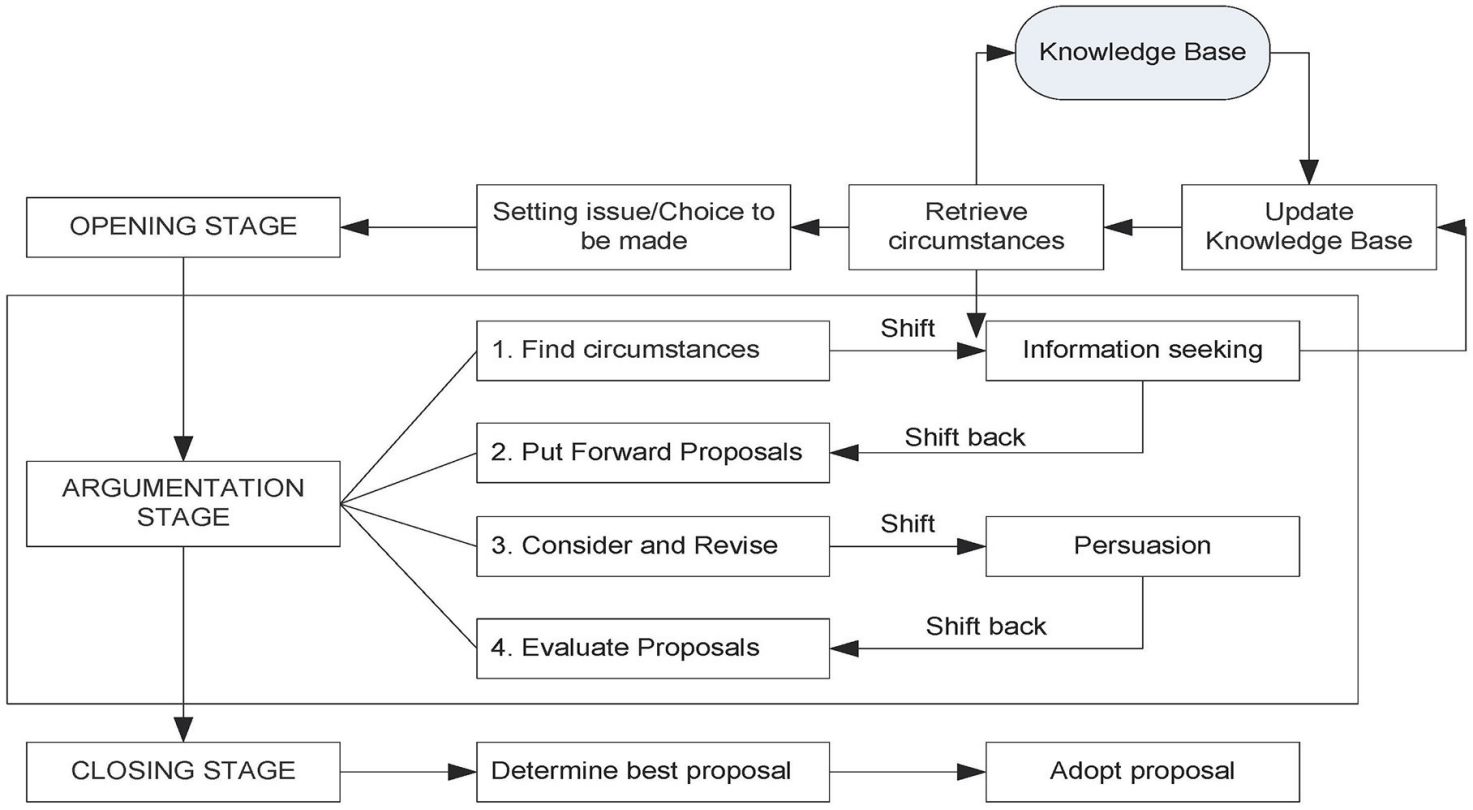
**The structure of the deliberation dialogue**.

The structure of deliberation dialogues is represented in Figure 2 (Walton et al., 2014).

As shown in Figure 2, deliberation dialogues usually develop in three stages: the opening stage, the argumentation stage and the closing stage. The stages of the dialogue do not correspond to phases in the structure of the medical encounter; their names refer to the development of the deliberative process and they are not meant to represent the chronological order in which stages appear in real-life interactions, even if they do represent the most logical order in which they should appear. In other words, they represent the ideal organization of an optimal deliberation dialogue.

Deliberation in this model is represented as a complex dialogue type, resulting from the combination and intersection between the persuasion and the information seeking dialogues.

In the opening stage, the parties share relevant information regarding the problem at issue. In this stage information is functional to the argumentative process that will follow, therefore it is crucial that parties share what they know about the problem, but also their preferences, values, circumstances that may in any way bear an import on the kind of solution that can be found. The aims of this stage remind of some of the suggestions for practice put forward by Fogarty (1997), in particular the indication to spend time with patients before the actual encounter, listening to their concerns of personal, non-medical nature (Fogarty, 1997, p. 1283). For Fogarty this procedure is aimed at keeping patients involved in the process of care, thereby limiting their perceptions of threat to their freedom. However, from a dialogical perspective, this is also a precious indication for the achievement of more effective deliberations. Indeed, proposals for therapeutic regimes or behaviors will be much more tailored to patients' actual conditions and abilities if providers are aware of them (in line with the suggestion to keep treatments as simple as possible, from the patient's point of view; to take into consideration patients' abilities to do certain things; and to find the best way to fit the prescribed regimens into patients' lifestyles).

The opening stage then gives way to the argumentation stage, which is the heart of deliberation. Here the parties start putting forward proposals for the solution of the problem, based on the previously shared information and on the shared goals of the interaction. The relevance of this stage in relation to the issue of reactance is very high. First, it is assumed that both parties should put forward at least one proposal to solve the problem. This is coherent with the indication to engage patients in the discussion in order to reach a negotiated regimen that is acceptable to both parties. Second, it is also foreseen by the model that if new information emerges during the argumentation stage this might imply that the parties revise their initial proposals. This is coherent with the suggestion to providers to be willing to make concessions and to offer more than one option for patients' choice.

Upon finding an option that is acceptable for both parties, the dialogue enters its closing stage. Here it is important that patients make explicit commitments to carrying out the chosen behavior or therapy. Providers' commitment to providing expert advice and the best options available are presupposed by the institutional context within which the interaction takes place.

This structure helps to clarify the role information and argumentation play within the deliberative process and their very close connection and interdependence. Moreover, at least at a theoretical level, it would seem that carrying out deliberations by following the structure and premises of the deliberation dialogue should prevent or limit the arousal of reactance. Indeed, the model foresees that both parties are fully engaged in the deliberative process at all stages, which basically realizes Fogarty's suggestion to keep patients involved in the process of care so as to limit reactance by softening the strength of threat.

Thus, theoretically speaking, this model of deliberation could be proposed to clinicians as a “deliberation protocol” that would allow to overcome sloppy or naïve—and thus, ineffective—realizations of deliberation. However, before discussing practical applications of the model, the role of information in relation to reactance should be discussed.

## The role of knowledge in relation to reactance

As already mentioned, patient education is discussed by Fogarty (1997, pp. 1284–1285) as a way to limit patient reactance and non-adherence. Moreover, the findings by Dillard and Shen (2005) show that information should be framed very carefully also depending on the potential strength of threat implied by certain health topics. Thus, the use of knowledge as a means to achieve adherence should be managed carefully. Indeed, more information can have the boomerang effect of increasing reactance, because by knowing more about their disease individuals are also more aware of possible further limitations on their freedom, thus behave in ways that try to prevent these limitations. Another problem with knowledge is that it may interfere with patients' Locus of Control: patients who wish to remain in control of their lives may feel excessively threatened by education programs that enhance their awareness of the many ways in which the disease and its treatments will “take control away” from them (Fogarty, 1997).

In order to better understand the role of information in the provider-patient encounter and in relation to the arousal of reactance, a general distinction that can be made is between information as the means to achieve patient education on the one hand, and as a component of the process of pragmatic argumentation on the other. This distinction can be useful because it allows to collocate the provision of information within two rather distinct dialogical processes, thus more easily identifying its role, potential and risks in relation to the arousal of reactance.

As a means to achieve patient education, the provision of information should be considered first of all from an institutional point of view. Indeed, the medical encounter is by definition an institutional context of interaction, with predefined roles, aims and norms that regulate the communicative exchange. Depending on the design of each healthcare system, these institutional elements may vary, but generally speaking providing information to patients regarding their health condition is considered to be one of the aims of the encounter. Therefore, providers cannot avoid this step and if knowledge can produce reactance, it is important to understand how it can be provided in a way that may limit or prevent its arousal.

Based on the considerations presented above referring to the boomerang effect of knowledge, it seems necessary to move away from a conception of education as mere transmission of information. In this respect, helpful insights may be gained from studies in the field of science education, in which it is shown experimentally that argumentative processes favor learning and understanding (Schwartz and Asterhan, 2010; Felton et al., 2015). More specifically, when students are given the task of “finding agreement” on an issue, they seem to be more willing to open up to different points of view and understandings of the problem. Moreover, argumentative practices seem fundamental for the achievement of conceptual change, i.e., a radical reframing of knowledge, which is often necessary in the case of lay beliefs and misrepresentations of health issues that patients often bring to the encounter and that impact on patients' expectations and on the ways they make decisions or form commitments. The “exercise” of using argumentative strategies for patient education in analogy to experiments with students could be done during group-work with patients, where peer-to-peer interaction may also favor the expression of personal beliefs. On the other hand, the “exercise” of reaching radical conceptual change regarding wrong beliefs or lay prejudices about diseases should probably be conducted during the one-to-one encounter with the physician.

In both cases, experimental interventions need to be carried out before it is possible to offer providers specific techniques for achieving patient education through argumentation.

In general, the role of information within a process of education is different from its role within a deliberative process. As discussed previously, in the latter situation information is relevant only if it informs the argumentative component of deliberation. This means that, if during the opening stage of deliberation, the provider realizes the patient has wrong beliefs or information about the disease, the dialogue may shift to an education dialogue and then shift back to the deliberation. However, the potential for threat to freedom seems different in the two cases: it may be higher during an education dialogue, because the provision of information entails expectations for further limitations to freedom; it should be lower during a deliberation dialogue, where patients should be fully involved in the decision, thus maintaining a fair degree of control over what is being decided and what they will have to commit to. An example of the first situation is provided by the following excerpt, from a consultation in a diabetes outpatient clinic (Bigi, 2014b). The patient (P) and the doctor (D) are discussing the patient's health situation and the patient starts asking questions about the correct choice of food:

P: what about beans, peas, can I eat those?

D: of course

P: but I noticed that they raise my glucose values

D: well, yes, you can eat them but appropriate quantities. So, for example, if you want to have pasta with beans you will add less pasta than when you have pasta with butter

P: also vegetable soup?

D: eh, of course because

P: if I make vegetable soup I noticed it [the glycemia] increases

D: vegetable soup, excellent question, it contains potatoes or carrots. Potatoes for example have a higher glycemic index so…

P: also carrots?

D: also carrots, but less than potatoes

P: I see…

This can be described as an education dialogue, because it has been triggered by a question from the patient, aimed at integrating a knowledge gap. The doctor replies by making examples of correct behaviors and by explaining the characteristics of different kinds of food. The doctor's explanations carry implications for the patient's behavior: if the patient is informed that potatoes contain more sugar than carrots, from now on he cannot just go on eating as many potatoes as he wants, because he can be held accountable for such behavior. In this sense, an education dialogue can contain a threat to freedom.

A different case is the one of the deliberation dialogue, like the following, also an excerpt from a consultation in a diabetes outpatient clinic (Bigi, 2014b). The doctor (D) has explained to the patient (P) that she really needs to be careful about her weight, because it could impact negatively on her diabetes. She starts a deliberation dialogue in which she invites the patient to decide how she wants to cope with the situation: D: ok, so from my point of view I can't suggest much. You are already taking strong medications for your diabetes, which means that if the three branches of a therapy are physical activity, medications and eating habits, I am already pretty high on medications. It would be better to act on the other two levels. Only one, both, a bit of both, you have to tell me. What do you think you can do?

P: I would like to…D: not I would like

P: no, I would like to, really…D: ok, what we would like is the ideal situation, it's perfection, but what is it that you can actually do at this point of your life?

P: I don't know what I will be able to do…

P's daughter: why don't you come to the gym with me, Mom? Three months?

P: ok, let's go, let's try…D: three months at the gym, ok then, 3 months at the gym, and we could add to that no restrictions on eating, but a very careful management of sweets

P: no…, look, I don't mind giving up sweets, but don't make me give up fruit

D: that's ok, I'm telling you, let's negotiate. Let's choose two things, 3 months at the gym, no sweets and you can have fruit. Let's try and see how it works, ok?

Also in this case, the doctor's opening turn contains an explanation that implies restrictions on the patient's freedom, but the deliberation dialogue that follows actively involves the patient in the process of decision making regarding which freedoms to give up and which to retain. In this particular case, the doctor is very good at allowing the patient to express her preferences, at the same time putting forward proposals for action that she can agree to or refuse. In this way, the patient retains control over the actions that are decided upon and she can freely decide what she wants to commit to.

So, while in the case of education, special attention should be paid to the way information is presented, in the case of deliberation it is the “procedure” that should be followed carefully in order to ensure patients' full participation, thereby limiting reactance. By “procedure” is meant the structure of the deliberation dialogue as described in Figure 2.

In both cases, the adoption of new strategies in the clinical practice entails a challenge at the institutional level of context, which is what we turn to in the following section.

## Introducing pro-engagement strategies in the clinical practice: a challenge for healthcare systems

As mentioned in the introductory section of the paper, the definition itself of patient engagement entails an opening to the wider context of the healthcare system within which providers and patients interact. The focus on very specific dialogical processes proposed in this paper does not imply a different perspective, quite the contrary.

The description of the model of the deliberation dialogue (see Figure 2) allows to visualize the components of this complex dialogical process, and to ponder their roles in relation to the achievement of the final goal of a participatory and collaborative decision (itself a pre-requisite for behavior change). In particular, the role of information has been discussed in relation to the aims of patient education and deliberation. The crucial role of argumentative strategies has been pointed out in relation to the prevention or softening of reactance, and the support to processes that may lead to full patient engagement.

The previous discussion has been conducted at a theoretical level and has proceeded from a top-down approach. The conclusions that can be drawn at this level are still in the form of hypotheses and will have to be confirmed through experiments and interventions. However, even at this theoretical level, the awareness of the optimal realization of crucial dialogical processes within the medical encounter allows some reflections regarding the setting in which such processes take place. In order to make the discussion more concrete, the Italian healthcare system will be used as an example, given the familiarity of the author with its structure (Bigi, 2012).

### The italian healthcare system

Italy's health care system as we know it today was officially born in 1978 (Centro di ricerca sulle amministrazioni pubbliche “V. Bachelet”, 2008, pp. 4–12). The system is founded on a principle of “universality,” which means that minimal levels of healthcare should be guaranteed to everyone. In coherence with this principle, medications and exams are mostly paid for by the healthcare system with resources collected through taxation. Citizens may be required to contribute for a smaller part to the expenses. As a consequence, the system is quite easily accessible, at least at the level of general practice, which is free and managed by single practitioners through appointments. Disadvantages of the system are that not all the 20 regions in which Italy is divided are able to manage resources optimally, which leads to bad imbalances in the provision of healthcare. Moreover, in case of economic crises, the central government may decide to cut the healthcare budget, which of course has an impact on the quality of care provided.

One important implication of healthcare being managed centrally is that all providers working for the public healthcare system are employees of the public administration and, as such, have the juridical status of “public officials.” This means that in their capacities as healthcare professionals they are the representatives of the central government, which delegates to them the safeguard of health intended as a public good (Costituzione della Repubblica italiana [Constitution of the Italian Republic], 2009, art.32). In this context, prescriptions and certificates signed by providers are public instruments, which, if improperly written or signed, can cause providers to undergo a penal trial.

In case of malpractice, the criteria that the judge uses to evaluate a healthcare professional's conduct are the ones of diligence and information. In the case of diligence, providers have to do everything and the best that is in their possibilities, having considered the situation of the patient (Bianca, 1965). In the case of information, professionals must provide all the relevant information to patients regarding the procedures they will undergo, along with potential risks and advantages. The Constitution (art. 32) states that nobody can be made to undergo a treatment unless they freely accept to. This leads to the obligation to provide and require explicit Informed Consent from patients, in all of the cases that entail procedures in which the patient is a passive subject (e.g., surgical operations). In the case of drug prescriptions, e.g., in outpatient clinics or in general practice, formal Informed Consent is not required because patients can always refuse treatment, but the obligation to provide thorough information remains. In any case, the only criterion that must guide providers' decisions is patients' quality of life and wellbeing.

### A challenge for the healthcare system

The description of the structure of the Italian healthcare system and of providers' juridical obligations reveals the degree of complexity of providers' task. As public officials, providers have an obligation to exercise their profession with diligence, which entails the obligation to information giving. On the other hand, in any circumstance patients are free (by law) to refuse or interrupt therapeutic regimens. The reasons for not wanting to take care of one's own health are not as improbable as they may seem. Incorrect information collected on the Internet, through acquaintances, via the mass media, may lead patients to become suspicious of certain treatments or to decide not to take any “chemical drugs.” Wrong beliefs regarding the definition of healthy food or healthy nutrition may lead patients to opt for unhealthy eating habits, thus damaging their health (see, Bigi and Pollaroli, 2016 for a discussion on this topic). The asymmetrical social roles predefined by the context of interaction may also play against the construction of trust between patients and providers, thus complicating even more the achievement of the institutional goals of patients' wellbeing.

Wrong information, badly informed beliefs, the tension created by misaligned expectancies related to each other's roles within the encounter may concur to create more opportunities for the arousal of reactance rather than patient engagement. Very recent data regarding patient non-adherence to treatments in Italy (Italian Medicines Agency, 2015) cannot be ascribed entirely to these causes, but certainly indicate that the problem exists and needs attention.

The discussion conducted in the preceding sections has pointed out the crucial role played by argumentation in both the process of patient education and of shared deliberation. In both cases, the potential for the reduction of reactance is very high and should be tested experimentally. However, in consideration of the institutional constraints presented in the previous section, there is one more reason to urge the system to innovate. Indeed, the obligation for providers to provide information to patients combined with findings on the high potential for a boomerang effect of information would suggest that providers be not only trained to provide information appropriately, but also be put in the conditions to do so. The suggestion of allowing more time and resources for trying out argumentative strategies in the context of patient education goes in this direction. Moreover, if patients are free to interrupt treatment at any time and providers cannot force them, it is also true that they can try to persuade them, if continuing treatment is good for patients' health. In this case too it is an issue of appropriate training in argumentation (Bigi, 2015 for a discussion on this point), but also of having the opportunity of spending time with patients, either personally or as a team.

As the issue of time is very common and cuts across national boundaries, a consideration regarding chronic conditions can be made. Indeed, the time for individual encounters is not very long and it is not possible to educate, motivate, and train patients to the use of devices, while listening to them and doing the paperwork required in the space of 15–18 min. However, chronic conditions have an “advantage,” which is that they last in time. The opportunity of meeting with patients on a regular basis for years, offers the possibility to distribute the various tasks over different encounters. One time it might be a priority to conduct a session of education on a certain topic, and the following time it might be more important to focus on motivating the patient.

Finally, as already argued by other scholars (Wagenaar, 2006), sometimes healthcare facilities should be designed more appropriately, in order to limit the perception of alienation (and thus, loss of freedom) in patients. Entering very complex buildings, with no clear signs and getting lost; interacting with dismissive or non-professional personnel; waiting for a long time in a stuffy corridor, with lots of people, while sitting on an uncomfortable chair: all this may not be conducive to collaborative and trusting relationships during the encounter.

Therefore, the challenge for any healthcare system, also in times of fewer resources and sustainability emergency, is not only to reduce budgets and cut on expenses, but also to rethink or restructure some crucial points in the system that hinder engagement and collaboration among all the actors in the system.

## Author contributions

The author confirms being the sole contributor of this work and approved it for publication.

## Funding

The author holds a 3-year grant funded by the Italian Ministry of Education, University and Research. Grant no.: RBFR13FQ5J. Project website: http://www.unicatt.it/healthyreasoning-eng.

### Conflict of interest statement

The author declares that the research was conducted in the absence of any commercial or financial relationships that could be construed as a potential conflict of interest.
